# Predicting the deforestation-trend under different carbon-prices

**DOI:** 10.1186/1750-0680-1-15

**Published:** 2006-12-06

**Authors:** Georg E Kindermann, Michael Obersteiner, Ewald Rametsteiner, Ian McCallum

**Affiliations:** 1 International Institute for Applied Systems Analysis (IIASA), Laxenburg, Austria; 2 University of Natural Resources and Applied Life Sciences (BOKU), Vienna, Austria; 3 Institute for Advanced Studies (IHS), Vienna, Austria

## Abstract

**Background:**

Global carbon stocks in forest biomass are decreasing by 1.1 Gt of carbon annually, owing to continued deforestation and forest degradation. Deforestation emissions are partly offset by forest expansion and increases in growing stock primarily in the extra-tropical north. Innovative financial mechanisms would be required to help reducing deforestation. Using a spatially explicit integrated biophysical and socio-economic land use model we estimated the impact of carbon price incentive schemes and payment modalities on deforestation. One payment modality is adding costs for carbon emission, the other is to pay incentives for keeping the forest carbon stock intact.

**Results:**

Baseline scenario calculations show that close to 200 mil ha or around 5% of todays forest area will be lost between 2006 and 2025, resulting in a release of additional 17.5 GtC. Today's forest cover will shrink by around 500 million hectares, which is 1/8 of the current forest cover, within the next 100 years. The accumulated carbon release during the next 100 years amounts to 45 GtC, which is 15% of the total carbon stored in forests today. Incentives of 6 US$/tC for vulnerable standing biomass payed every 5 year will bring deforestation down by 50%. This will cause costs of 34 billion US$/year. On the other hand a carbon tax of 12 $/tC harvested forest biomass will also cut deforestation by half. The tax income will, if enforced, decrease from 6 billion US$ in 2005 to 4.3 billion US$ in 2025 and 0.7 billion US$ in 2100 due to decreasing deforestation speed.

**Conclusion:**

Avoiding deforestation requires financial mechanisms that make retention of forests economically competitive with the currently often preferred option to seek profits from other land uses. Incentive payments need to be at a very high level to be effective against deforestation. Taxes on the other hand will extract budgetary revenues from the regions which are already poor. A combination of incentives and taxes could turn out to be a viable solution for this problem. Increasing the value of forest land and thereby make it less easily prone to deforestation would act as a strong incentive to increase productivity of agricultural and fuelwood production, which could be supported by revenues generated by the deforestation tax.

## Background

Deforestation is considered the second largest source of greenhouse gas (GHG) emissions amounting to an estimated 2 gigatonnes of carbon (GtC) per annum over the last decade [1]. It is a persistent problem. The UN Food and Agriculture Organization, in its recently released most comprehensive assessment of forests ever, puts deforestation at about 12.9 mil. ha per year [2]. At the same time, forest planting, landscape restoration and natural expansion of forests reduce the net loss of forest area. Net change in forest area in the period 2000–2005 is estimated at -7.3 million hectares per year [2]. This reduces the annual GHG emissions to an estimated 1.1 GtC. In comparison, 7.3 GtC were emitted in 2003 by using fossil energy sources [3].

Deforestation has been difficult to tackle by governments, as its drivers are complex and many land uses yield higher revenues than those from forested land. Some see climate policy as a new opportunity to effectively reduce a major source of greenhouse gases and biodiversity loss as well as to increase incomes of many people in rural areas whose livelihood depends on forests. The implementation of measures avoiding deforestation would require innovative financial mechanisms in the context of global climate policies. In this paper we study the potential magnitude of effects of different financial mechanisms to help reduce deforestation, using a modeling approach.

To estimate the impact of financial incentives, to reduce deforestation and assuming profit maximizing behavior, we calculate differences in net present value of different land uses using a spatially explicit integrated biophysical and socio-economic land use model. Key model parameters, such as agricultural land use and production, population growth, deforestation and forest product consumption rates were calibrated against historical rates. Land use changes are simulated in the model as a decision based on a difference between net present value of income from production on agricultural land versus net present value of income from forest products. Assuming fixed technology, the model calculates for each 0.5° grid cell the net present value difference between agricultural and forest land-uses in one-year time steps. When carbon market prices, transferred through a financial mechanism, balance out differences between the net present value of agricultural land and forest-related income, it is assumed, consistent with profit maximising behavior, that deforestation is avoided.

The net present value difference of forest versus other land uses can be balanced out through two mechanisms. One is to reduce the difference by adding costs to conversion through taxing emissions from deforestation, e. g. through a land clearance tax and wood sales taxes. The other is to enhance the value of the existing forest by financial support when keeping the forest carbon stock, to be paid in certain time intervals. In both cases the value of forest carbon stock would be pegged to carbon market prices. The modeling results for different hypothetical tax or subsidy levels show the potential magnitude of avoided deforestation through financial incentive or disincentive mechanisms. The model results are annual, spatially explicit estimates of the forest area and biomass development from 2000 to 2100, with particular focus on the period 2006 to 2025.

## Results and discussion

### Baseline deforestation 2000–2100 and effects of financial mechanisms aiming at cutting emissions in half

**Figure 1:**
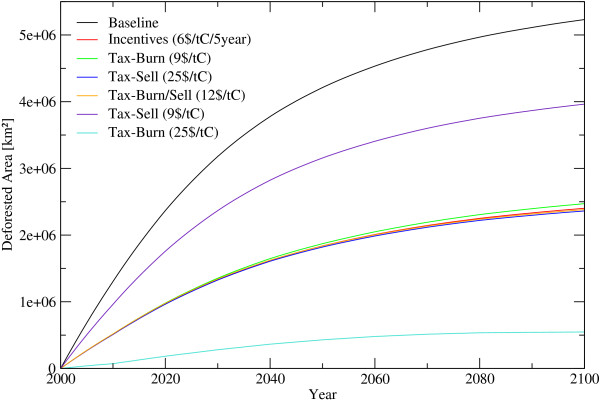
**Deforested Area until 2100**. Deforested Area under alternative assumptions. *Incentives*... Periodic payments for standing biomass, *Tax*... Payments for harvesting wood, *Burn*... felled wood is burned immediately, *Sell*... harvested wood is soled, *Burn/Sell*... share of the wood will be burned the other part soled.

Baseline scenario calculations (i.e. a carbon price of 0 US$/tC is assumed) show that close to 200 mil ha or around 5% of todays forest area will be lost between 2006 and 2025, resulting in a release of additional 17.5 GtC to the atmospheric carbon pool. The baseline deforestation speed is decreasing over time, which is caused by a decreasing forest area in regions with hight deforestation pressure. In the year 2025 the annual deforested area decreases to 8.2 million hectares, compared to 12.9 million hectares in 2005. By the year 2100 deforestation rates decline to some 1.1 million hectares. According to the base line scenario, today's forest cover will shrink by around 500 million hectares or by more than 1/8 within the next 100 years (figure 1).

**Figure 2:**
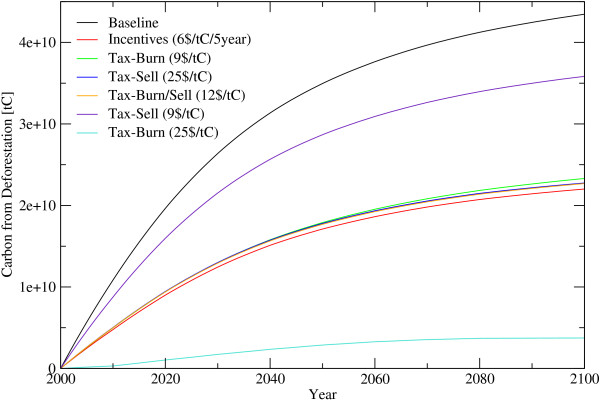
**Released Carbon from Deforestation until 2100**. Released carbon from deforestation under alternative assumptions. *Incentives*... Periodic payments for standing biomass, *Tax*... Payments for harvesting wood, *Burn*... felled wood is burned immediately, *Sell*... harvested wood is soled, *Burn/Sell*... share of the wood will be burned the other part soled.

**Figure 3:**
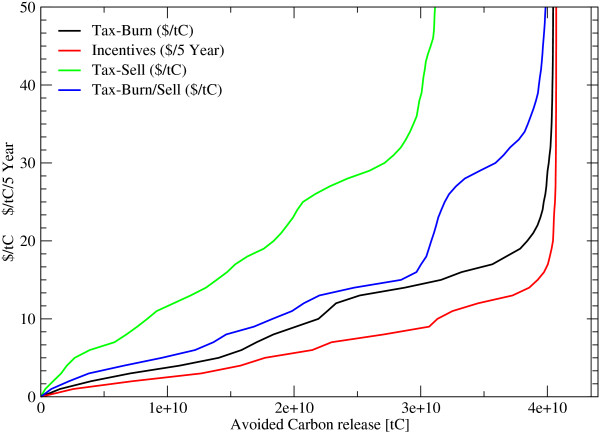
**Avoided Carbon releases under different Carbon prices during the next 100 years**. *Incentives*... Periodic payments for standing biomass, *Tax*... Payments for harvesting wood, *Burn*... felled wood is burned immediately, *Sell*... harvested wood is soled, *Burn/Sell*... share of the wood will be burned the other part soled.

**Figure 4:**
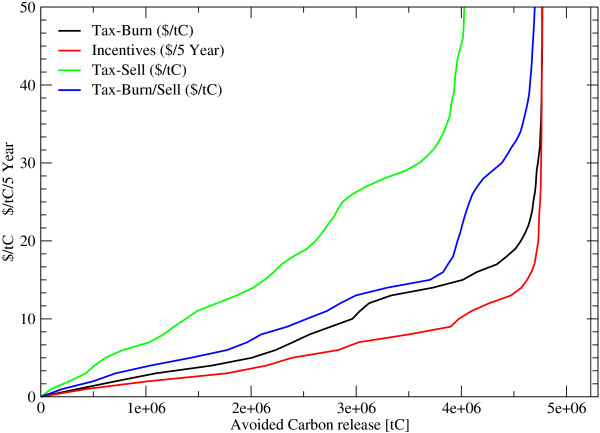
**Saved Forest Area under different Carbon prices during the next 100 years**. *Incentives*... Periodic payments for standing biomass, *Tax*... Payments for harvesting wood, *Burn*... felled wood is burned immediately, *Sell*... harvested wood is soled, *Burn/Sell*... share of the wood will be burned the other part soled.

Carbon emissions from deforestation in 2005 is 1.1 GtC/year and decreases to 0.68 GtC/year in 2025 and further to 0.09 GtC/year in 2100. The accumulated carbon release during the next 100 years amounts to 45 GtC which is 15% of the total carbon stored in forests today. To bring deforestation down by 50%, incentives of 6 US$/tC/5 year or a land clearance tax of between 9 US$/tC and 25 US$/tC would be necessary, depending whether the harvested wood is burned on the spot (e. g. slash-and-burn agriculture) or sold. In the latter case, a higher carbon tax of up to 25 US$/tC is necessary to effectively reduce incentives to deforest, to a degree that cuts overall global deforestation by 50%. If the wood is further used and converted into products, only 18% of the biomass could be saved by a carbon price of 9 US$/tC, caused by the compensating effect of an income by selling wood and a longer time-period for releasing carbon. On the other hand, if the carbon price is 25$/tC and the wood is assumed to be slash burned, the reduction of deforestation calculated to be 91% (figure 1 and 2). On a first sight it seems, that incentive payments might be more effective, than taxation. However, incentives payment contracts have to be renewed every 5 year for the actual standing biomass and the change of biomass has to be known to detect a breach of the contract, while a deforestation tax will be payed once for the harvested biomass once detected by targeted earth observation systems (see figure 3 and 4). In the latter, transactions costs for implementing avoided deforestation are small.

The assumption, that either only slash burn or all wood will be sold is unrealistic. Thus, a scenario where Latin America has 90% slash burn and 10% selling, Africa 50% slash burned and 50% selling and in the remaining area 10% slash burned and 90% selling, was examined. Under such scenario assumptions a carbon tax of 12 $/tC will cut deforestation in half. Also the assumption, that a carbon price will stay constant over time may not be close to reality but it can be used to see the long-term influence of a given carbon price.

We differentiate between the following cases:

**Baseline: **Introducing no carbon price.

**Incentives: **Introducing a carbon price which will be payed periodic for the carbon stored in the standing forest biomass.

**All**: Payments are done, without considering the effectiveness of the payment, in all regions.

**Region: **Payments are done in regions where the payments protect forest against deforestation.

**Affected: **Payments are done for forests where the payments protect them against deforestation.

**Tax: **Introducing a carbon price which has to be paid for releasing the stored carbon to the atmosphere.

**Burn: **All wood will be burned immediately.

**Sell: **All harvested wood will be sold.

**Burn/Sell: **A share of the wood will be burned and the other part sold.

### Costs and revenues under different carbon prices

**Figure 5:**
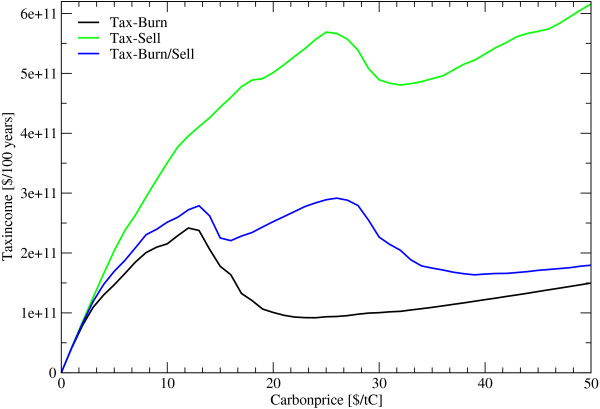
**Income under different Carbon Prices**. *Tax*... Payments for harvesting wood, *Burn*... felled wood is burned immediately, *Sell*... harvested wood is soled, *Burn/Sell*... share of the wood will be burned the other part soled.

The effectiveness of introducing a carbon price to influence deforestation decisions depends largely on the levels set for carbon prices, apart from considerations of political feasibility and implementability. Low prices have little impact on deforestation rates. During the 21^*st *^century carbon tax schemes of 9 US$/tC for slash burn and 25 US$/tC for situations when removed wood enters a harvested wood products pool (HWP) would generate some 2 to 5.7 billion US$/year respectively when emissions from deforestation are to be cut in half. For the variant of 12 US$/tC, with regionally differentiated slash burn and HWP assumptions, the average annual income for the next 100 years are calculated to be around 2.7 billion US$. These tax revenues decrease dramatically over time mainly due to the declining baseline deforestation rate. Tax revenues are computed to be 6 billion US$ in 2005, 4.3 billion US$ in 2025 and 0.7 billion US$ in 2100. This indicates the magnitudes and their temporal change of funds generated from a deforestation tax scheme aiming at a 50% emission reduction (figure 5 and 7).

**Figure 6:**
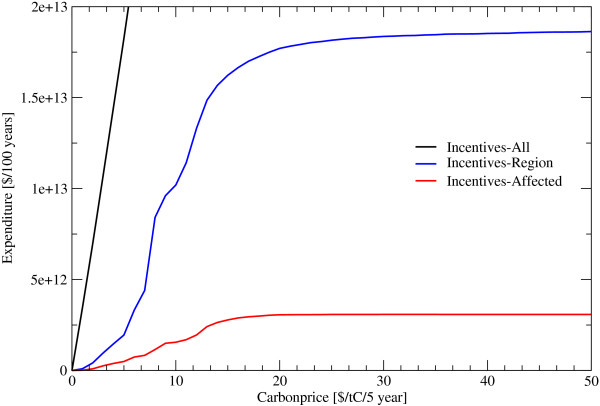
**Expenditure under different Carbon Prices**. *Incentives*... Periodic payments for standing biomass, *All*... Payments are done, without considering the effectiveness of the payment, in all regions, *Region*... Payments are done in regions where the payments protect forest against deforestation, *Affected*... Payments are done for forests where the payments protect them against deforestation.

In the alternative incentive scheme, the amount of funds necessary, is depending on the strategy of payments, either increasing, staying constant or decreasing over time. If incentives are paid only for those forest areas that are about to be deforested, and with a global target of cutting deforestation by 50%, a minimum payment of 6 US$/tC/5 year or 0.24 billion US$ in 2006 would be required. This amount rises to some 1.2 billion US$ in 2010, 4.1 billion US$ in 2025 and 10 billion US$ in 2100 caused by the increasing area of saved forest area. As precise information of forests about to be deforested is absent, incentive payment schemes would have to focus on regions under deforestation pressure. Given that incentives are only spent on regions of 0.5° × 0.5° where they can effectively reduce deforestation in an amount that they will balance out the income difference between forests and alternative land use up the 6 US$/tC/5 year, this would come at a cost of 34 billion US$/year (figure 6 and 8). It should be noted that the tax applies only on places currently deforested while the subsidy applies to larger areas depending on how far it is in practice possible to restrict the subsidy to vulnerable areas. All figures above are intentionally free of transaction costs. Transaction costs would inter alia include expenditure for protecting the forests against illegal logging by force and expenditures monitoring small scale forest degregation. Governance issues such as corruption and risk adjustment, depending on the country are, however, considered in the analysis to the extent possible.

**Figure 7:**
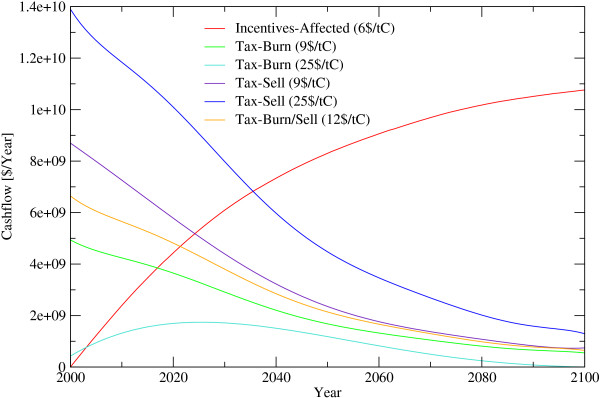
**Cash flow until 2100 for different Carbon Prices**. *Incentives*... Periodic payments for standing biomass, *Tax*... Payments for harvesting wood, *Affected*... Payments are done for forests where the payments protect them against deforestation, *Burn*... felled wood is burned immediately, *Sell*... harvested wood is soled, *Burn/Sell*... share of the wood will be burned the other part soled.

**Figure 8:**
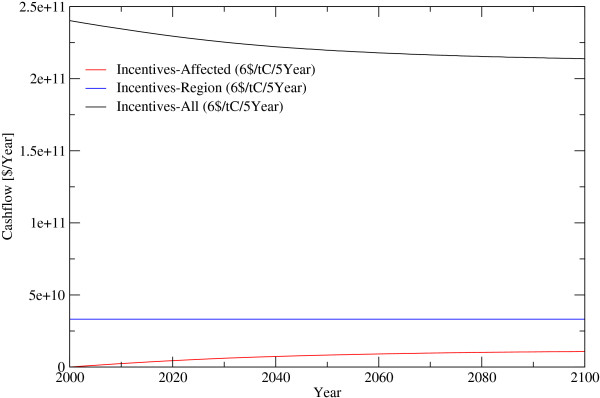
**Expenditure until 2100 for different Incentive payment Strategies**. *Incentives*... Periodic payments for standing biomass, *All*... Payments are done, without considering the effectiveness of the payment, in all regions, *Region*... Payments are done in regions where the payments protect forest against deforestation, *Affected*... Payments are done for forests where the payments protect them against deforestation.

### Regional effects of carbon prices on deforestation

Sources of deforestation in the model are expansion of agriculture and buildup areas as well as from unsustainable timber harvesting operations impairing sufficient reforestation. Deforestation results from many pressures, both local and international. While the more direct causes are rather well established as being agricultural expansion, infrastructure extension and wood extraction, indirect drivers of deforestation are made up of a complex web of interlinked and place-specific factors. There is large spatially differentiated heterogeneity of deforestation pressures. Within a forest-agriculture mosaic, forests are under high deforestation pressure unless they are on sites which are less suitable for agriculture (swamp, slope, altitude). Closed forests at the frontier to agriculture land are also under a high deforestation pressure while forest beyond this frontier are under low pressure as long as they are badly attainable. The model was build to capture such heterogeneity in deforestation pressures.

**Figure 9:**
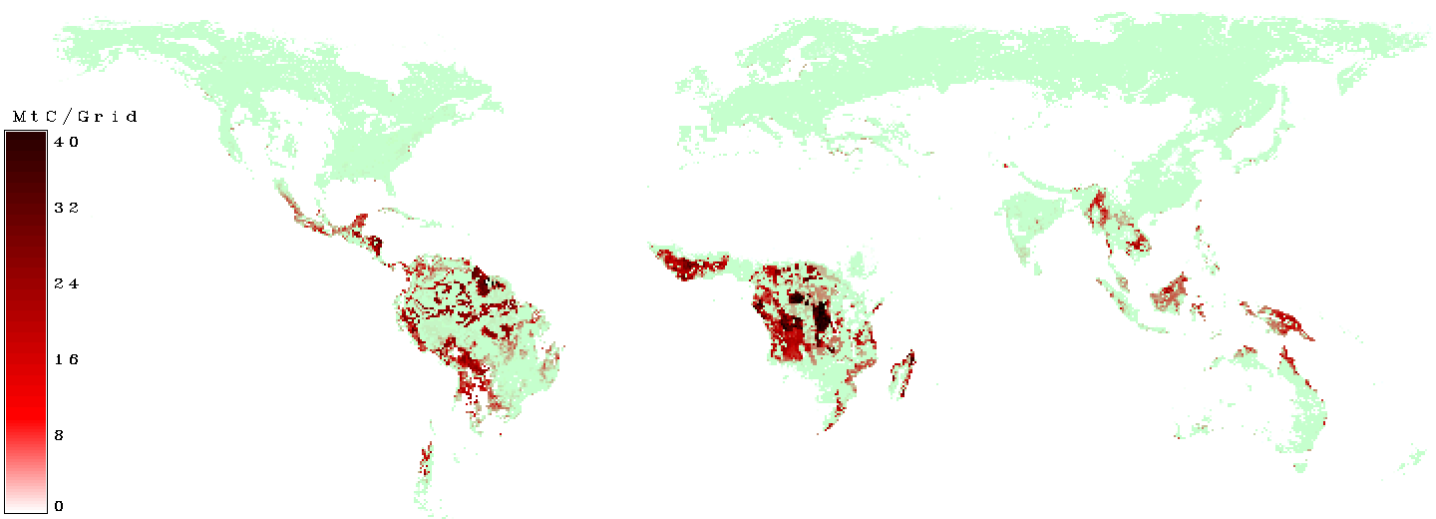
**Removed Biomass without a carbon price**. Green areas show grids where nowadays forests can be found. Red areas indicate grids where deforestation will occur in a scenario without carbon prices.

**Figure 10:**
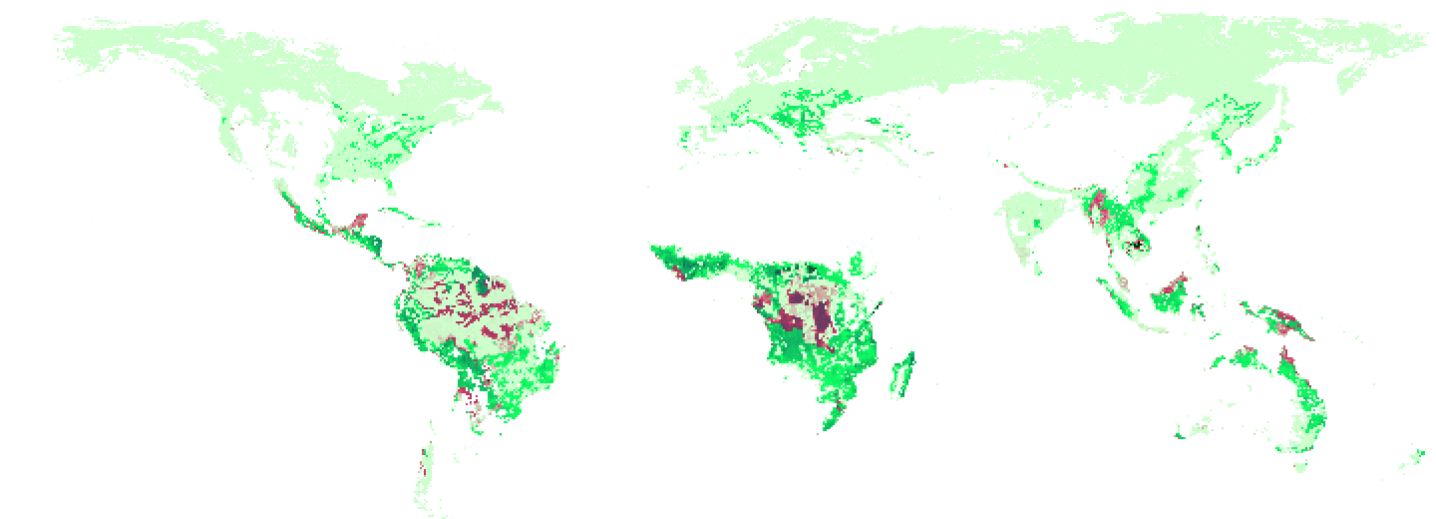
**Saved Biomass by 12$/tC (Burn Sell)**. Light green show grids where nowadays forests can be found. Dark green areas indicate grids where forest biomass can be saved by introducing a carbon price of 12$/tC compared to the baseline scenario. Red ares indicate grids where there will still be deforestation.

Figure 9 shows that the model predicts deforestation to continue at the frontier to agricultural land and in areas which are easly accessible. Trans-frontier forests are also predicted to be deforested due to their relative accessibility and agricultural suitablility. Forests in mosaic lands continue to be under strong pressure. Figure 10 illustrates the geography of carbon saved at a carbon tax of 12 US$/tC compared to biomass lost through deforestation. Under this scenario deforestation is maily occurring in clusters, which are sometimes surrounded by forests (e.g. Central Africa) or are concentrated along a line (Amazon). The geography of the remaining deforestation pattern indicates that large areas are prevented from deforestation at the frontier by the 12 US$/tC tax. The remaining emissions from deforestation are explained mainly by their accessibility and favourable agricultural suitability.

## Conclusion

Avoiding deforestation requires financial mechanisms that make retention of forests economically competitive with the currently often preferred option to seek profits from other land uses. According to the model calculations, even relatively low carbon incentives of around 6 $/tC/5 year, paid for forest carbon stock retention or carbon taxes of 12 $/tC would suffice to effectively cut emissions from deforestation by half. Taxes revenues would bring about annual income of US$6 bn in 2005 to US$0.7 bn in 2100. The financial means required for incentives are estimated to range from US$3 bn to US$ 200 bn per year, depending on the design of the avoided deforestation policy. Our scenario, where incentives are payed in regions where deforestation will appear and the payment has an effect, estimates the necessary funds to cut emissions from deforestation in half in the magnitude of some US$ 33 bn per year, without including costs for transaction, observation and illegal logging protection. Increasing the value of forest land and thereby make it less easily prone to deforestation would act as a strong incentive to increase productivity of agricultural and fuelwood production.

## Methods

The model is based mainly on the global afforestation model of [4] and calculates the net present value of forestry with equation (1 – 16) and the net present value of agriculture with equation (17 – 20). Main drivers for the net present value of forestry are income from carbon sequestration, wood increment, rotation period length, discount rates, planting costs and wood prices. Main drivers for the net present value of agriculture on current forest land are population density, agricultural suitability and risk adjusted discount rates.

These two values are compared against each other and deforestation is subsequently predicted to occur when the agricultural value exceeds the forest value by a certain margin. When the model comes to the result, that deforestation occurs, the speed of deforestation was constraint by estimates given by equation (24). The speed of deforestation is a function of sub-grid forest share, agricultural suitability, population density and economic wealth of the country.

All symbols in the following equations are explained in the section "Abbreviations".

### Net present value of forestry

The net present value of forestry is determined by the planting costs, the harvestable wood volume, the wood-price and benefits from carbon sequestration.

For existing forests which are assumed to be under active managment the net present value of forestry given multiple rotations (*F*_*i*_) over the simulation horizon is calculated from the net present value for one rotation (*f*_*i*_) (equation 1). This is calculated by taking into account the planting costs (*cp*_*i*_) at the begin of the rotation period and the income from selling the harvested wood (*pw*_*i*_·*V*_*i*_) at the end of the rotation period. Also the benefits from carbon sequestration are included denoted as (*B*_*i*_).

The planting costs (eq. 3) are calculated by multiplying the planting costs of the reference country (*cp*_*ref*_) with a price index (*px*_*i*_) and a factor which describes the share of natural regeneration (*pr*_*i*_). The ratio of plantation to natural regeneration is assumed to increase with increasing yield for the respective forests (eq. 4). The price index (eq. 5) is calculated using the purchasing power parity of the respective countries. The stumpage wood price (eq. 6) is calculated from the harvest cost free income range of wood in the reference country. This price is at the lower bound when the population density is low and the forest share is high and at the higher bound when the population density is high and the forest share is low. The price is also multiplied with a price index converting the price range from the reference country to the examined country. The population-density and forest-share was standardized between 1 and 10 by using equation (7) and equation (8) respectively.

The harvested volume (*V*_*i*_) is calculated by multiplying the mean annual increment (*MAI*_*i*_) with the rotation period length (*R*_*i*_) accounting for harvesting losses (eq. 9).

The rotation period length (eq. 10) depends on the yield. Fast growing stands have a short and slow growing sites a long rotation length. In this study the rotation length is in the range between 5 and 140 years.

The mean annual increment (eq. 11) is calculated by multiplying the estimated carbon uptake (*ω*_*i*_) and a transformation factor which brings the carbon weight to a wood volume (*C*2*W*_*i*_). The carbon uptake (*ω*_*i*_) is calculated by multiplying the net primary production (*NPP*_*i*_) with a factor describing the share of carbon uptake from the net primary production (eq. 12).

The benefits of carbon sequestration (eq. 13) are calculated by discounting the annual income from additional carbon sequestration and subtracting the expenses incurred from harvesting operations and silvicultural production. At the end of a rotation period the harvested carbon is still stored in harvested wood products and will come back to atmosphere with a delay. This is considered in the factor (*θ*_*i*_) which shares the harvested wood volume to short and long living products(eq. 14).

The effective carbon price represents the benefit which will directly go to the forest owner. In equation (16) a factor describing the percentage of the transaction cost free carbon price is used. A factor *leak*_*i *_is calculated as the average of the percentile rank from "political stability", "government effectiveness" and "control of corruption" [5].

Fi=fi⋅[1−(1+r)−Ri]−1     (1)
 MathType@MTEF@5@5@+=feaafiart1ev1aaatCvAUfKttLearuWrP9MDH5MBPbIqV92AaeXatLxBI9gBaebbnrfifHhDYfgasaacH8akY=wiFfYdH8Gipec8Eeeu0xXdbba9frFj0=OqFfea0dXdd9vqai=hGuQ8kuc9pgc9s8qqaq=dirpe0xb9q8qiLsFr0=vr0=vr0dc8meaabaqaciaacaGaaeqabaqabeGadaaakeaacqWGgbGrdaWgaaWcbaGaemyAaKgabeaakiabg2da9iabdAgaMnaaBaaaleaacqWGPbqAaeqaaOGaeyyXICTaei4waSLaeGymaeJaeyOeI0IaeiikaGIaeGymaeJaey4kaSIaemOCaiNaeiykaKYaaWbaaSqabeaacqGHsislcqWGsbGudaWgaaadbaGaemyAaKgabeaaaaGccqGGDbqxdaahaaWcbeqaaiabgkHiTiabigdaXaaakiaaxMaacaWLjaWaaeWaaeaacqaIXaqmaiaawIcacaGLPaaaaaa@4880@

*f*_*i *_= -*cp*_*i *_+ *pw*_*i*_·*V*_*i *_+ *B*_*i *_    (2)

*cp*_*i *_= *cp*_*ref*_·*pr*_*i*_·*px*_*i *_    (3)

pri={0MAIi<3(MAIi−3)/63≤MAIi≤91MAIi>9     (4)
 MathType@MTEF@5@5@+=feaafiart1ev1aaatCvAUfKttLearuWrP9MDH5MBPbIqV92AaeXatLxBI9gBaebbnrfifHhDYfgasaacH8akY=wiFfYdH8Gipec8Eeeu0xXdbba9frFj0=OqFfea0dXdd9vqai=hGuQ8kuc9pgc9s8qqaq=dirpe0xb9q8qiLsFr0=vr0=vr0dc8meaabaqaciaacaGaaeqabaqabeGadaaakeaacqWGWbaCcqWGYbGCdaWgaaWcbaGaemyAaKgabeaakiabg2da9maaceqabaqbaeaabmGaaaqaaiabicdaWaqaaiabd2eanjabdgeabjabdMeajnaaBaaaleaacqWGPbqAaeqaaOGaeyipaWJaeG4mamdabaGaeiikaGIaemyta0KaemyqaeKaemysaK0aaSbaaSqaaiabdMgaPbqabaGccqGHsislcqaIZaWmcqGGPaqkcqGGVaWlcqaI2aGnaeaacqaIZaWmcqGHKjYOcqWGnbqtcqWGbbqqcqWGjbqsdaWgaaWcbaGaemyAaKgabeaakiabgsMiJkabiMda5aqaaiabigdaXaqaaiabd2eanjabdgeabjabdMeajnaaBaaaleaacqWGPbqAaeqaaOGaeyOpa4JaeGyoaKdaaaGaay5EaaGaaCzcaiaaxMaadaqadaqaaiabisda0aGaayjkaiaawMcaaaaa@5B20@

pxi=PPPiPPPref     (5)
 MathType@MTEF@5@5@+=feaafiart1ev1aaatCvAUfKttLearuWrP9MDH5MBPbIqV92AaeXatLxBI9gBaebbnrfifHhDYfgasaacH8akY=wiFfYdH8Gipec8Eeeu0xXdbba9frFj0=OqFfea0dXdd9vqai=hGuQ8kuc9pgc9s8qqaq=dirpe0xb9q8qiLsFr0=vr0=vr0dc8meaabaqaciaacaGaaeqabaqabeGadaaakeaacqWGWbaCcqWG4baEdaWgaaWcbaGaemyAaKgabeaakiabg2da9maalaaabaGaemiuaaLaemiuaaLaemiuaa1aaSbaaSqaaiabdMgaPbqabaaakeaacqWGqbaucqWGqbaucqWGqbaudaWgaaWcbaGaemOCaiNaemyzauMaemOzaygabeaaaaGccaWLjaGaaCzcamaabmaabaGaeGynaudacaGLOaGaayzkaaaaaa@42CC@

pwi=pwmin−pwmax−pwmin99+pwmax−pwmin99⋅SPd⋅SNFs⋅pxi     (6)
 MathType@MTEF@5@5@+=feaafiart1ev1aaatCvAUfKttLearuWrP9MDH5MBPbIqV92AaeXatLxBI9gBaebbnrfifHhDYfgasaacH8akY=wiFfYdH8Gipec8Eeeu0xXdbba9frFj0=OqFfea0dXdd9vqai=hGuQ8kuc9pgc9s8qqaq=dirpe0xb9q8qiLsFr0=vr0=vr0dc8meaabaqaciaacaGaaeqabaqabeGadaaakeaacqWGWbaCcqWG3bWDdaWgaaWcbaGaemyAaKgabeaakiabg2da9iabdchaWjabdEha3naaBaaaleaaieGacqWFTbqBcqWFPbqAcqWFUbGBaeqaaOGaeyOeI0YaaSaaaeaacqWGWbaCcqWG3bWDdaWgaaWcbaGae8xBa0Mae8xyaeMae8hEaGhabeaakiabgkHiTiabdchaWjabdEha3naaBaaaleaacqWFTbqBcqWFPbqAcqWFUbGBaeqaaaGcbaGaeGyoaKJaeGyoaKdaaiabgUcaRmaalaaabaGaemiCaaNaem4DaC3aaSbaaSqaaiab=1gaTjab=fgaHjab=Hha4bqabaGccqGHsislcqWGWbaCcqWG3bWDdaWgaaWcbaGae8xBa0Mae8xAaKMae8NBa4gabeaaaOqaaiabiMda5iabiMda5aaacqGHflY1cqWGtbWucqWGqbaucqWGKbazcqGHflY1cqWGtbWucqWGobGtcqWGgbGrcqWGZbWCcqGHflY1cqWGWbaCcqWG4baEdaWgaaWcbaGaemyAaKgabeaakiaaxMaacaWLjaWaaeWaaeaacqaI2aGnaiaawIcacaGLPaaaaaa@7579@

SPd={1+Pd⋅9100Pd≤10010Pd>100     (7)
 MathType@MTEF@5@5@+=feaafiart1ev1aaatCvAUfKttLearuWrP9MDH5MBPbIqV92AaeXatLxBI9gBaebbnrfifHhDYfgasaacH8akY=wiFfYdH8Gipec8Eeeu0xXdbba9frFj0=OqFfea0dXdd9vqai=hGuQ8kuc9pgc9s8qqaq=dirpe0xb9q8qiLsFr0=vr0=vr0dc8meaabaqaciaacaGaaeqabaqabeGadaaakeaacqWGtbWucqWGqbaucqWGKbazcqGH9aqpdaGabeqaauaabmqaciaaaeaacqaIXaqmcqGHRaWkdaWcaaqaaiabdcfaqjabdsgaKjabgwSixlabiMda5aqaaiabigdaXiabicdaWiabicdaWaaaaeaacqWGqbaucqWGKbazcqGHKjYOcqaIXaqmcqaIWaamcqaIWaamaeaacqaIXaqmcqaIWaamaeaacqWGqbaucqWGKbazcqGH+aGpcqaIXaqmcqaIWaamcqaIWaamaaaacaGL7baacaWLjaGaaCzcamaabmaabaGaeG4naCdacaGLOaGaayzkaaaaaa@4FEA@

*SNFs *= 1 + (1 - *Fs*) * 9     (8)

*V*_*i *_= *MAI*_*i*_·*R*_*i*_·(1 - *HL*_*i*_)     (9)

Ri={5MAIi>180/10600−|MAIi−6|⋅50MAIi103≤MAIi≤18010140MAIi<10/3     (10)
 MathType@MTEF@5@5@+=feaafiart1ev1aaatCvAUfKttLearuWrP9MDH5MBPbIqV92AaeXatLxBI9gBaebbnrfifHhDYfgasaacH8akY=wiFfYdH8Gipec8Eeeu0xXdbba9frFj0=OqFfea0dXdd9vqai=hGuQ8kuc9pgc9s8qqaq=dirpe0xb9q8qiLsFr0=vr0=vr0dc8meaabaqaciaacaGaaeqabaqabeGadaaakeaacqWGsbGudaWgaaWcbaGaemyAaKgabeaakiabg2da9maaceqabaqbaeaabmGaaaqaaiabiwda1aqaaiabd2eanjabdgeabjabdMeajnaaBaaaleaacqWGPbqAaeqaaOGaeyOpa4JaeGymaeJaeGioaGJaeGimaaJaei4la8IaeGymaeJaeGimaadabaWaaSaaaeaacqaI2aGncqaIWaamcqaIWaamcqGHsislcqGG8baFcqWGnbqtcqWGbbqqcqWGjbqsdaWgaaWcbaGaemyAaKgabeaakiabgkHiTiabiAda2iabcYha8jabgwSixlabiwda1iabicdaWaqaaiabd2eanjabdgeabjabdMeajnaaBaaaleaacqWGPbqAaeqaaaaaaOqaamaalaaabaGaeGymaeJaeGimaadabaGaeG4mamdaaiabgsMiJkabd2eanjabdgeabjabdMeajnaaBaaaleaacqWGPbqAaeqaaOGaeyizIm6aaSaaaeaacqaIXaqmcqaI4aaocqaIWaamaeaacqaIXaqmcqaIWaamaaaabaGaeGymaeJaeGinaqJaeGimaadabaGaemyta0KaemyqaeKaemysaK0aaSbaaSqaaiabdMgaPbqabaGccqGH8aapcqaIXaqmcqaIWaamcqGGVaWlcqaIZaWmaaaacaGL7baacaWLjaGaaCzcamaabmaabaGaeGymaeJaeGimaadacaGLOaGaayzkaaaaaa@75BA@

*MAI*_*i *_= *ω*_*i*_·*C*2*W *    (11)

*ω*_*i *_= *NPP*_*i*_·*CU *    (12)

Bi=epci⋅ωi⋅(1−bi)⋅{r−1⋅[1−(1+r)−Ri]−Ri⋅(1−θi)⋅(1+r)−Ri}     (13)
 MathType@MTEF@5@5@+=feaafiart1ev1aaatCvAUfKttLearuWrP9MDH5MBPbIqV92AaeXatLxBI9gBaebbnrfifHhDYfgasaacH8akY=wiFfYdH8Gipec8Eeeu0xXdbba9frFj0=OqFfea0dXdd9vqai=hGuQ8kuc9pgc9s8qqaq=dirpe0xb9q8qiLsFr0=vr0=vr0dc8meaabaqaciaacaGaaeqabaqabeGadaaakeaacqWGcbGqdaWgaaWcbaGaemyAaKgabeaakiabg2da9iabdwgaLjabdchaWjabdogaJnaaBaaaleaacqWGPbqAaeqaaOGaeyyXICncciGae8xYdC3aaSbaaSqaaiabdMgaPbqabaGccqGHflY1cqGGOaakcqaIXaqmcqGHsislcqWGIbGydaWgaaWcbaGaemyAaKgabeaakiabcMcaPiabgwSixlabcUha7jabdkhaYnaaCaaaleqabaGaeyOeI0IaeGymaedaaOGaeyyXICTaei4waSLaeGymaeJaeyOeI0IaeiikaGIaeGymaeJaey4kaSIaemOCaiNaeiykaKYaaWbaaSqabeaacqGHsislcqWGsbGudaWgaaadbaGaemyAaKgabeaaaaGccqGGDbqxcqGHsislcqWGsbGudaWgaaWcbaGaemyAaKgabeaakiabgwSixlabcIcaOiabigdaXiabgkHiTiab=H7aXnaaBaaaleaacqWGPbqAaeqaaOGaeiykaKIaeyyXICTaeiikaGIaeGymaeJaey4kaSIaemOCaiNaeiykaKYaaWbaaSqabeaacqGHsislcqWGsbGudaWgaaadbaGaemyAaKgabeaaaaGccqGG9bqFcaWLjaGaaCzcamaabmaabaGaeGymaeJaeG4mamdacaGLOaGaayzkaaaaaa@7919@

θi=(1−decllp⋅fracllpdecllp+r−decslp⋅fracslpdecslp+r)⋅(1−fracsb)+(1−fracsb)∗fracsb     (14)
 MathType@MTEF@5@5@+=feaafiart1ev1aaatCvAUfKttLearuWrP9MDH5MBPbIqV92AaeXatLxBI9gBaebbnrfifHhDYfgasaacH8akY=wiFfYdH8Gipec8Eeeu0xXdbba9frFj0=OqFfea0dXdd9vqai=hGuQ8kuc9pgc9s8qqaq=dirpe0xb9q8qiLsFr0=vr0=vr0dc8meaabaqaciaacaGaaeqabaqabeGadaaakeaaiiGacqWF4oqCdaWgaaWcbaGaemyAaKgabeaakiabg2da9iabcIcaOiabigdaXiabgkHiTmaalaaabaGaemizaqMaemyzauMaem4yam2aaSbaaSqaaiabdYgaSjabdYgaSjabdchaWbqabaGccqGHflY1cqWGMbGzcqWGYbGCcqWGHbqycqWGJbWydaWgaaWcbaGaemiBaWMaemiBaWMaemiCaahabeaaaOqaaiabdsgaKjabdwgaLjabdogaJnaaBaaaleaacqWGSbaBcqWGSbaBcqWGWbaCaeqaaOGaey4kaSIaemOCaihaaiabgkHiTmaalaaabaGaemizaqMaemyzauMaem4yam2aaSbaaSqaaiabdohaZjabdYgaSjabdchaWbqabaGccqGHflY1cqWGMbGzcqWGYbGCcqWGHbqycqWGJbWydaWgaaWcbaGaem4CamNaemiBaWMaemiCaahabeaaaOqaaiabdsgaKjabdwgaLjabdogaJnaaBaaaleaacqWGZbWCcqWGSbaBcqWGWbaCaeqaaOGaey4kaSIaemOCaihaaiabcMcaPiabgwSixlabcIcaOiabigdaXiabgkHiTiabdAgaMjabdkhaYjabdggaHjabdogaJnaaBaaaleaacqWGZbWCcqWGIbGyaeqaaOGaeiykaKIaey4kaSIaeiikaGIaeGymaeJaeyOeI0IaemOzayMaemOCaiNaemyyaeMaem4yam2aaSbaaSqaaiabdohaZjabdkgaIbqabaGccqGGPaqkcqGHxiIkcqWGMbGzcqWGYbGCcqWGHbqycqWGJbWydaWgaaWcbaGaem4CamNaemOyaigabeaakiaaxMaacaWLjaWaaeWaaeaacqaIXaqmcqaI0aanaiaawIcacaGLPaaaaaa@9C98@

*frac*_*slp *_= 1 - *frac*_*llp *_    (15)

*epc*_*i *_= *pc*_*i*_·*leak*_*i *_    (16)

### Net present value of agriculture

The net present value of agriculture (*A*_*i*_) is calculated with a two-factor Cobb-Douglas production function (equation 17). It depends on the agriculture suitability and the population density. A high agriculture suitability and a high population density causes high agricultural values. The value ranges between a given minimum and a maximum land price. The parameters *a*_*i *_and *γ*_*i *_determine the relative importance of the agriculture suitability and the population density and *ν*_*i *_determines the price level for land. The agriculture suitability and the population density are normalized between 1 and 10.

Ai=νi⋅SAgSiαi⋅SPdiγi     (17)
 MathType@MTEF@5@5@+=feaafiart1ev1aaatCvAUfKttLearuWrP9MDH5MBPbIqV92AaeXatLxBI9gBaebbnrfifHhDYfgasaacH8akY=wiFfYdH8Gipec8Eeeu0xXdbba9frFj0=OqFfea0dXdd9vqai=hGuQ8kuc9pgc9s8qqaq=dirpe0xb9q8qiLsFr0=vr0=vr0dc8meaabaqaciaacaGaaeqabaqabeGadaaakeaacqWGbbqqdaWgaaWcbaGaemyAaKgabeaakiabg2da9GGaciab=17aUnaaBaaaleaacqWGPbqAaeqaaOGaeyyXICTaem4uamLaemyqaeKaem4zaCMaem4uam1aa0baaSqaaiabdMgaPbqaaiab=f7aHnaaBaaameaacqWGPbqAaeqaaaaakiabgwSixlabdofatjabdcfaqjabdsgaKnaaDaaaleaacqWGPbqAaeaacqWFZoWzdaWgaaadbaGaemyAaKgabeaaaaGccaWLjaGaaCzcamaabmaabaGaeGymaeJaeG4naCdacaGLOaGaayzkaaaaaa@4EC4@

SAgSi={10AgSi≥0.51+9⋅AgS/0.5AgSi<0.5     (18)
 MathType@MTEF@5@5@+=feaafiart1ev1aaatCvAUfKttLearuWrP9MDH5MBPbIqV92AaeXatLxBI9gBaebbnrfifHhDYfgasaacH8akY=wiFfYdH8Gipec8Eeeu0xXdbba9frFj0=OqFfea0dXdd9vqai=hGuQ8kuc9pgc9s8qqaq=dirpe0xb9q8qiLsFr0=vr0=vr0dc8meaabaqaciaacaGaaeqabaqabeGadaaakeaacqWGtbWucqWGbbqqcqWGNbWzcqWGtbWudaWgaaWcbaGaemyAaKgabeaakiabg2da9maaceqabaqbaeaabiGaaaqaaiabigdaXiabicdaWaqaaiabdgeabjabdEgaNjabdofatnaaBaaaleaacqWGPbqAaeqaaOGaeyyzImRaeGimaaJaeiOla4IaeGynaudabaGaeGymaeJaey4kaSIaeGyoaKJaeyyXICTaemyqaeKaem4zaCMaem4uamLaei4la8IaeGimaaJaeiOla4IaeGynaudabaGaemyqaeKaem4zaCMaem4uam1aaSbaaSqaaiabdMgaPbqabaGccqGH8aapcqaIWaamcqGGUaGlcqaI1aqnaaaacaGL7baacaWLjaGaaCzcamaabmaabaGaeGymaeJaeGioaGdacaGLOaGaayzkaaaaaa@5AC5@

αi=ln⁡(PLmax)−ln⁡(PLmin)2⋅ln⁡(10)     (19)
 MathType@MTEF@5@5@+=feaafiart1ev1aaatCvAUfKttLearuWrP9MDH5MBPbIqV92AaeXatLxBI9gBaebbnrfifHhDYfgasaacH8akY=wiFfYdH8Gipec8Eeeu0xXdbba9frFj0=OqFfea0dXdd9vqai=hGuQ8kuc9pgc9s8qqaq=dirpe0xb9q8qiLsFr0=vr0=vr0dc8meaabaqaciaacaGaaeqabaqabeGadaaakeaaiiGacqWFXoqydaWgaaWcbaGaemyAaKgabeaakiabg2da9maalaaabaGagiiBaWMaeiOBa4MaeiikaGIaemiuaaLaemitaW0aaSbaaSqaaGqaciab+1gaTjab+fgaHjab+Hha4bqabaGccqGGPaqkcqGHsislcyGGSbaBcqGGUbGBcqGGOaakcqWGqbaucqWGmbatdaWgaaWcbaGae4xBa0Mae4xAaKMae4NBa4gabeaakiabcMcaPaqaaiabikdaYiabgwSixlGbcYgaSjabc6gaUjabcIcaOiabigdaXiabicdaWiabcMcaPaaacaWLjaGaaCzcamaabmaabaGaeGymaeJaeGyoaKdacaGLOaGaayzkaaaaaa@565C@

*γ*_*i *_= *α*_*i *_    (20)

### Decision of deforestation

The deforestation decision is expressed by equation (21). It compares the agricultural and forestry net present values corrected by values for deforestation and carbon sequestration. For the deforestation decision the amount of removed biomass from the forest is an important variable. The agricultural value needed for deforestation increases with the amount of timber sales and its concomitant flow to the HWP pool. On the other hand the agriculture value will be decreased by the amount of released carbon to the atmosphere. This mechanism is expressed by a deforestation value (*DV*_*i*_, eq. 22). The model also allows for compensation of ancillary benefits from forests. This additional income is modeled either as a periodical income or a one time payment and will increase the forestry value by (*IP*_*i*_). If it is a periodic payment it has to be discounted, which has been done in equation (23).

Defor={YesAi+DVi>Fi⋅Hi+IPi∧not ProtectedNoAi+DVi≤Fi⋅Hi+IPi∨Protected     (21)
 MathType@MTEF@5@5@+=feaafiart1ev1aaatCvAUfKttLearuWrP9MDH5MBPbIqV92AaeXatLxBI9gBaebbnrfifHhDYfgasaacH8akY=wiFfYdH8Gipec8Eeeu0xXdbba9frFj0=OqFfea0dXdd9vqai=hGuQ8kuc9pgc9s8qqaq=dirpe0xb9q8qiLsFr0=vr0=vr0dc8meaabaqaciaacaGaaeqabaqabeGadaaakeaacqqGebarcqqGLbqzcqqGMbGzcqqGVbWBcqqGYbGCcqGH9aqpdaGabeqaauaabeqaciaaaeaacqqGzbqwcqqGLbqzcqqGZbWCaeaafaqaaeGabaaabaGaemyqae0aaSbaaSqaaiabdMgaPbqabaGccqGHRaWkcqWGebarcqWGwbGvdaWgaaWcbaGaemyAaKgabeaakiabg6da+iabdAeagnaaBaaaleaacqWGPbqAaeqaaOGaeyyXICTaemisaG0aaSbaaSqaaiabdMgaPbqabaGccqGHRaWkcqWGjbqscqWGqbaudaWgaaWcbaGaemyAaKgabeaaaOqaaiabgEIizlabb6gaUjabb+gaVjabbsha0jabbccaGiabbcfaqjabbkhaYjabb+gaVjabbsha0jabbwgaLjabbogaJjabbsha0jabbwgaLjabbsgaKbaaaeaacqqGobGtcqqGVbWBaeaafaqaaeGabaaabaGaemyqae0aaSbaaSqaaiabdMgaPbqabaGccqGHRaWkcqWGebarcqWGwbGvdaWgaaWcbaGaemyAaKgabeaakiabgsMiJkabdAeagnaaBaaaleaacqWGPbqAaeqaaOGaeyyXICTaemisaG0aaSbaaSqaaiabdMgaPbqabaGccqGHRaWkcqWGjbqscqWGqbaudaWgaaWcbaGaemyAaKgabeaaaOqaaiabgIIiAlabbcfaqjabbkhaYjabb+gaVjabbsha0jabbwgaLjabbogaJjabbsha0jabbwgaLjabbsgaKbaaaaaacaGL7baacaWLjaGaaCzcamaabmaabaGaeGOmaiJaeGymaedacaGLOaGaayzkaaaaaa@8B31@

DVi=BMi⋅{pwi⋅C2W⋅(1−HLi)−epci⋅[(1+r)⋅(fracllp⋅decllpdecllp+r+fracslp⋅decslpdecslp+r)⋅(1−fracsb)+fracsb]}     (22)
 MathType@MTEF@5@5@+=feaafiart1ev1aaatCvAUfKttLearuWrP9MDH5MBPbIqV92AaeXatLxBI9gBaebbnrfifHhDYfgasaacH8akY=wiFfYdH8Gipec8Eeeu0xXdbba9frFj0=OqFfea0dXdd9vqai=hGuQ8kuc9pgc9s8qqaq=dirpe0xb9q8qiLsFr0=vr0=vr0dc8meaabaqaciaacaGaaeqabaqabeGadaaakeaacqWGebarcqWGwbGvdaWgaaWcbaGaemyAaKgabeaakiabg2da9iabdkeacjabd2eannaaBaaaleaacqWGPbqAaeqaaOGaeyyXIC9aaiWabeaacqWGWbaCcqWG3bWDdaWgaaWcbaGaemyAaKgabeaakiabgwSixlabdoeadjabikdaYiabdEfaxjabgwSixlabcIcaOiabigdaXiabgkHiTiabdIeaijabdYeamnaaBaaaleaacqWGPbqAaeqaaOGaeiykaKIaeyOeI0IaemyzauMaemiCaaNaem4yam2aaSbaaSqaaiabdMgaPbqabaGccqGHflY1daWadaqaaiabcIcaOiabigdaXiabgUcaRiabdkhaYjabcMcaPiabgwSixpaabmaabaWaaSaaaeaacqWGMbGzcqWGYbGCcqWGHbqycqWGJbWydaWgaaWcbaGaemiBaWMaemiBaWMaemiCaahabeaakiabgwSixlabdsgaKjabdwgaLjabdogaJnaaBaaaleaacqWGSbaBcqWGSbaBcqWGWbaCaeqaaaGcbaGaemizaqMaemyzauMaem4yam2aaSbaaSqaaiabdYgaSjabdYgaSjabdchaWbqabaGccqGHRaWkcqWGYbGCaaGaey4kaSYaaSaaaeaacqWGMbGzcqWGYbGCcqWGHbqycqWGJbWydaWgaaWcbaGaem4CamNaemiBaWMaemiCaahabeaakiabgwSixlabdsgaKjabdwgaLjabdogaJnaaBaaaleaacqWGZbWCcqWGSbaBcqWGWbaCaeqaaaGcbaGaemizaqMaemyzauMaem4yam2aaSbaaSqaaiabdohaZjabdYgaSjabdchaWbqabaGccqGHRaWkcqWGYbGCaaaacaGLOaGaayzkaaGaeyyXICTaeiikaGIaeGymaeJaeyOeI0IaemOzayMaemOCaiNaemyyaeMaem4yam2aaSbaaSqaaiabdohaZjabdkgaIbqabaGccqGGPaqkcqGHRaWkcqWGMbGzcqWGYbGCcqWGHbqycqWGJbWydaWgaaWcbaGaem4CamNaemOyaigabeaaaOGaay5waiaaw2faaaGaay5Eaiaaw2haaiaaxMaacaWLjaWaaeWaaeaacqaIYaGmcqaIYaGmaiaawIcacaGLPaaaaaa@BC16@

IPi=(BMi+BMPi)⋅pcai⋅(r+1)fri(r+1)fri−1     (23)
 MathType@MTEF@5@5@+=feaafiart1ev1aaatCvAUfKttLearuWrP9MDH5MBPbIqV92AaeXatLxBI9gBaebbnrfifHhDYfgasaacH8akY=wiFfYdH8Gipec8Eeeu0xXdbba9frFj0=OqFfea0dXdd9vqai=hGuQ8kuc9pgc9s8qqaq=dirpe0xb9q8qiLsFr0=vr0=vr0dc8meaabaqaciaacaGaaeqabaqabeGadaaakeaacqWGjbqscqWGqbaudaWgaaWcbaGaemyAaKgabeaakiabg2da9iabcIcaOiabdkeacjabd2eannaaBaaaleaacqWGPbqAaeqaaOGaey4kaSIaemOqaiKaemyta0Kaemiuaa1aaSbaaSqaaiabdMgaPbqabaGccqGGPaqkcqGHflY1cqWGWbaCcqWGJbWycqWGHbqydaWgaaWcbaGaemyAaKgabeaakiabgwSixpaalaaabaGaeiikaGIaemOCaiNaey4kaSIaeGymaeJaeiykaKYaaWbaaSqabeaacqWGMbGzcqWGYbGCdaWgaaadbaGaemyAaKgabeaaaaaakeaacqGGOaakcqWGYbGCcqGHRaWkcqaIXaqmcqGGPaqkdaahaaWcbeqaaiabdAgaMjabdkhaYnaaBaaameaacqWGPbqAaeqaaaaakiabgkHiTiabigdaXaaacaWLjaGaaCzcamaabmaabaGaeGOmaiJaeG4mamdacaGLOaGaayzkaaaaaa@6072@

There exist several ways of how financial transfers can be handled. Two mechanisms are realized in equation (21). One is to pay the forest owner to avert from the deforestation, the other is to introduce a carbon price that the forest owner gets money by storing carbon and paying for releasing it. The introduction of a carbon price focuses the money transfer to the regions where a change in biomass takes place. Payments to avoid emissions from deforestation can be transfered to cover all of the globe's forests, target to large "deforestation regions" or individual grids.

### Deforestation rate

Once the principle deforestation decision has been made for a particular grid cell (i.e. the indicator variable *Defor*_*i *_= 1) the actual area to be deforested within the respective grid is to be determined. This is done by the auxiliary equation (24 – 25) computing the decrease in forest share. We model the deforestation rate within a particular grid as a function of its share of forest cover, agricultural suitability, population density and gross domestic product. The coefficients *c*_1 _to *c*_6 _were estimated with a generalized linear model of the quasibinomial family with a logit link. Values significant at a level of 5% were taken and are shown in table 1. The parameters of the regression model were estimated using R [6]. The value of c_0 _was determined upon conjecture and directly influences the maximum possible deforestation rate. For our scenarios the maximum possible deforestation is set to 5% of the total land area per year. That means, a 0.5° × 0.5° grid covered totally with forests can not be deforested in a shorter time period than 20 years.

**Table 1:** Coefficients for equation (25) – Deforestation speed

Coef	Estimate	Std. Error	Pr(> |*t*|)	
*c*_0_	0.05	-	-	
*c*_1_	-1.799e+00	4.874e-01	0.000310	***
*c*_2_	-2.200e-01	9.346e-02	0.019865	*
*c*_3_	-1.663e-01	5.154e-02	0.001529	**
*c*_4_	4.029e-02	1.712e-02	0.019852	*
*c*_5_	-5.305e-04	1.669e-04	0.001789	**
*c*_6_	-1.282e-04	3.372e-05	0.000206	***

Fdeci={0Defor=NoFsiFtdeci>Fsi∧Defor=YesFtdeciFtdeci≤Fsi∧Defor=Yes     (24)
 MathType@MTEF@5@5@+=feaafiart1ev1aaatCvAUfKttLearuWrP9MDH5MBPbIqV92AaeXatLxBI9gBaebbnrfifHhDYfgasaacH8akY=wiFfYdH8Gipec8Eeeu0xXdbba9frFj0=OqFfea0dXdd9vqai=hGuQ8kuc9pgc9s8qqaq=dirpe0xb9q8qiLsFr0=vr0=vr0dc8meaabaqaciaacaGaaeqabaqabeGadaaakeaacqWGgbGrcqWGKbazcqWGLbqzcqWGJbWydaWgaaWcbaGaemyAaKgabeaakiabg2da9maaceqabaqbaeaabmGaaaqaaiabicdaWaqaaiabbseaejabbwgaLjabbAgaMjabb+gaVjabbkhaYjabg2da9iabb6eaojabb+gaVbqaaiabdAeagjabdohaZnaaBaaaleaacqWGPbqAaeqaaaGcbaGaemOrayKaemiDaqNaemizaqMaemyzauMaem4yam2aaSbaaSqaaiabdMgaPbqabaGccqGH+aGpcqWGgbGrcqWGZbWCdaWgaaWcbaGaemyAaKgabeaakiabgEIizlabbseaejabbwgaLjabbAgaMjabb+gaVjabbkhaYjabg2da9iabbMfazjabbwgaLjabbohaZbqaaiabdAeagjabdsha0jabdsgaKjabdwgaLjabdogaJnaaBaaaleaacqWGPbqAaeqaaaGcbaGaemOrayKaemiDaqNaemizaqMaemyzauMaem4yam2aaSbaaSqaaiabdMgaPbqabaGccqGHKjYOcqWGgbGrcqWGZbWCdaWgaaWcbaGaemyAaKgabeaakiabgEIizlabbseaejabbwgaLjabbAgaMjabb+gaVjabbkhaYjabg2da9iabbMfazjabbwgaLjabbohaZbaaaiaawUhaaiaaxMaacaWLjaWaaeWaaeaacqaIYaGmcqaI0aanaiaawIcacaGLPaaaaaa@86A2@

Ftdeci={0Fsi=0∨AgSi=0xiFsi>0∧AgSi>0     (25)
 MathType@MTEF@5@5@+=feaafiart1ev1aaatCvAUfKttLearuWrP9MDH5MBPbIqV92AaeXatLxBI9gBaebbnrfifHhDYfgasaacH8akY=wiFfYdH8Gipec8Eeeu0xXdbba9frFj0=OqFfea0dXdd9vqai=hGuQ8kuc9pgc9s8qqaq=dirpe0xb9q8qiLsFr0=vr0=vr0dc8meaabaqaciaacaGaaeqabaqabeGadaaakeaacqWGgbGrcqWG0baDcqWGKbazcqWGLbqzcqWGJbWydaWgaaWcbaGaemyAaKgabeaakiabg2da9maaceqabaqbaeaabiGaaaqaaiabicdaWaqaaiabdAeagjabdohaZnaaBaaaleaacqWGPbqAaeqaaOGaeyypa0JaeGimaaJaeyikIOTaemyqaeKaem4zaCMaem4uam1aaSbaaSqaaiabdMgaPbqabaGccqGH9aqpcqaIWaamaeaacqWG4baEdaWgaaWcbaGaemyAaKgabeaaaOqaaiabdAeagjabdohaZnaaBaaaleaacqWGPbqAaeqaaOGaeyOpa4JaeGimaaJaey4jIKTaemyqaeKaem4zaCMaem4uam1aaSbaaSqaaiabdMgaPbqabaGccqGH+aGpcqaIWaamaaaacaGL7baacaWLjaGaaCzcamaabmaabaGaeGOmaiJaeGynaudacaGLOaGaayzkaaaaaa@5D35@

xi=c01+e−(c1+c2Fsi+c3AgSi+c4⋅Pdi+c5⋅Pdi2+c6⋅GDPi)     (26)
 MathType@MTEF@5@5@+=feaafiart1ev1aaatCvAUfKttLearuWrP9MDH5MBPbIqV92AaeXatLxBI9gBaebbnrfifHhDYfgasaacH8akY=wiFfYdH8Gipec8Eeeu0xXdbba9frFj0=OqFfea0dXdd9vqai=hGuQ8kuc9pgc9s8qqaq=dirpe0xb9q8qiLsFr0=vr0=vr0dc8meaabaqaciaacaGaaeqabaqabeGadaaakeaacqWG4baEdaWgaaWcbaGaemyAaKgabeaakiabg2da9maalaaabaGaem4yam2aaSbaaSqaaiabicdaWaqabaaakeaacqaIXaqmcqGHRaWkcqWGLbqzdaahaaWcbeqaaiabgkHiTiabcIcaOiabdogaJnaaBaaameaacqaIXaqmaeqaaSGaey4kaSYaaSaaaeaacqWGJbWydaWgaaadbaGaeGOmaidabeaaaSqaaiabdAeagjabdohaZnaaBaaameaacqWGPbqAaeqaaaaaliabgUcaRmaalaaabaGaem4yam2aaSbaaWqaaiabiodaZaqabaaaleaacqWGbbqqcqWGNbWzcqWGtbWudaWgaaadbaGaemyAaKgabeaaaaWccqGHRaWkcqWGJbWydaWgaaadbaGaeGinaqdabeaaliabgwSixlabdcfaqjabdsgaKnaaBaaameaacqWGPbqAaeqaaSGaey4kaSIaem4yam2aaSbaaWqaaiabiwda1aqabaWccqGHflY1cqWGqbaucqWGKbazdaqhaaadbaGaemyAaKgabaGaeGOmaidaaSGaey4kaSIaem4yam2aaSbaaWqaaiabiAda2aqabaWccqGHflY1cqWGhbWrcqWGebarcqWGqbaudaWgaaadbaGaemyAaKgabeaaliabcMcaPaaaaaGccaWLjaGaaCzcamaabmaabaGaeGOmaiJaeGOnaydacaGLOaGaayzkaaaaaa@6F6F@

The deforestation rates (*Ft*_*dec*_) were taken from [2], where the forest area from 1990, 2000 and 2005 for each country was given. For the estimation of the model parameters the area difference between 1990 and 2005 was used to infer the deforestation rate. All values which showed an increase of the forest area have been set to 0, because the model should only predict the deforestation. Countries with an increasing forest area have a deforestation rate of 0. It should be mentioned that the change rate is based on the total land area in the grid i and not on the current forest area.

By using *c*_2_/*F*_*s *_the model can only be used on grid's where there is some share of forest. This makes sense, because on places where there is no forest, no deforestation can appear. The model will only be usable on grids where forests occur. Therefore, for parameterization, the average agricultural suitability and the population density of a country are also only taken from grids which indicate forest cover.

### Development of forest share

After calculating the deforestation rate, the forest share has to be updated each year with equation (27) assuring that the forest share stays within the permissible range of 0–1.

Fsi,year={fsxi,yearfsxi,year≤1−(Buli+Crli)1−(Buli+Crli)fsxi,year>1−(Buli+Crli)     (27)
 MathType@MTEF@5@5@+=feaafiart1ev1aaatCvAUfKttLearuWrP9MDH5MBPbIqV92AaeXatLxBI9gBaebbnrfifHhDYfgasaacH8akY=wiFfYdH8Gipec8Eeeu0xXdbba9frFj0=OqFfea0dXdd9vqai=hGuQ8kuc9pgc9s8qqaq=dirpe0xb9q8qiLsFr0=vr0=vr0dc8meaabaqaciaacaGaaeqabaqabeGadaaakeaacqWGgbGrcqWGZbWCdaWgaaWcbaGaemyAaKMaeiilaWIaemyEaKNaemyzauMaemyyaeMaemOCaihabeaakiabg2da9maaceqabaqbaeaabiGaaaqaaiabdAgaMjabdohaZjabdIha4naaBaaaleaacqWGPbqAcqGGSaalcqWG5bqEcqWGLbqzcqWGHbqycqWGYbGCaeqaaaGcbaGaemOzayMaem4CamNaemiEaG3aaSbaaSqaaiabdMgaPjabcYcaSiabdMha5jabdwgaLjabdggaHjabdkhaYbqabaGccqGHKjYOcqaIXaqmcqGHsislcqGGOaakcqWGcbGqcqWG1bqDcqWGSbaBdaWgaaWcbaGaemyAaKgabeaakiabgUcaRiabdoeadjabdkhaYjabdYgaSnaaBaaaleaacqWGPbqAaeqaaOGaeiykaKcabaGaeGymaeJaeyOeI0IaeiikaGIaemOqaiKaemyDauNaemiBaW2aaSbaaSqaaiabdMgaPbqabaGccqGHRaWkcqWGdbWqcqWGYbGCcqWGSbaBdaWgaaWcbaGaemyAaKgabeaakiabcMcaPaqaaiabdAgaMjabdohaZjabdIha4naaBaaaleaacqWGPbqAcqGGSaalcqWG5bqEcqWGLbqzcqWGHbqycqWGYbGCaeqaaOGaeyOpa4JaeGymaeJaeyOeI0IaeiikaGIaemOqaiKaemyDauNaemiBaW2aaSbaaSqaaiabdMgaPbqabaGccqGHRaWkcqWGdbWqcqWGYbGCcqWGSbaBdaWgaaWcbaGaemyAaKgabeaakiabcMcaPaaaaiaawUhaaiaaxMaacaWLjaWaaeWaaeaacqaIYaGmcqaI3aWnaiaawIcacaGLPaaaaaa@935E@

*fsx*_*i, year *_= *Fs*_*i, year *- 1 _- *F*_*i, dec *_    (28)

### Aboveground carbon in forest biomass

The model describes the area covered by forests on a certain grid. It can also describe the forest biomass if the average biomass on a grid is known and the assumption was made, that the biomass in forests on the grid is proportional to the forest area.

For this reason a global carbon map of aboveground carbon in forest biomass, was created, based on country values from [2]. By dividing the given total carbon, for each country, with the forest area of the country, the average biomass per hectare can be calculated. Now the assumption was made, that the stocking biomass per hectare on sites with a higher productivity is higher than on sites with a low productivity. Not for every country with forests [2] gives values of the stocking biomass. So a regression, describing the relation between tC/ha and NPP, was calculated and the biomass of grids of missing countries have been estimated to obtain a complete global forest biomass map.

### Simulations

In the simulations the effect of different carbon-prices and/or incentives, for keeping forest, have been tested. The simulation period started in the year 2000 and ends in 2100. The decision, whether deforestation takes place or not and how fast it goes on, was done in one year time steps. Scenario drivers, available on coarser time resolution (e.g. population density), have been interpolated linearly between the given years.

Outputs of the simulations are trajectoria of forest cover, changes in carbon stocks of forests, and financial resources required to cut emissions from deforestation under varying scenario assumptions.

### Data

The model uses several sources of input data some available for each grid, some by country aggregates and others are global. The data supporting the values in table 2 are known for each grid. Some of the values are also available for time series.

**Table 2:** Spatial dataset available on a 0.5° × 0.5° grid

Value	Year	Source
Land area	2000	[11]
Country	2000	[12]
NPP	-	[10]
Population density	1990 – 2015	[13]
Population density	1990 – 2100	[14]
GDP	1990 – 2100	[14]
Buildup	2010 – 2080	[15]
Crop	2010 – 2080	[15]
Protected	2004	[16]
Agriculture suitability	2002	[17]
Biomass	2005	Self
Forest area	2000	[11]

Beside the datasets, available at grid level, the purchasing power parity PPP [7] from 1975–2003, the discount rates [8] for 2004, the corruption in 2005 [5] and the fraction of long living products for the time span 2000–2005 [2] are available for each country (table 3).

**Table 3:** Country level values

	Source
Discount rate	[8]
Fraction of long living products	[2]
Corruption	[5]
PPP	[7]

The values of table 4 are used globally. Monetary values are transformed for each country with their price index. Brazil was taken as the price-reference country as described in [8] and [9].

**Table 4:** Global values

Baseline	0.1
Decay rate long	ln(2)/20
Decay rate short	0.5
Factor carbon uptake	0.5
Frequency of incentives payment	5 years
tC to m^3^	4
Harvest losses	0.3
Hurdle	1.5
Maximum rotation interval	140 years
Minimum rotation interval	5 years
Planting costs	800 $/ha
Carbon price	0–50 $/tC
Carbon price incentives	0–50 $/tC
Minimum Land price	200 $/ha
Maximum Land price	900 $/ha
Minimum wood price	5$/ha
Maximum wood price	35$/ha

**Figure 11:**
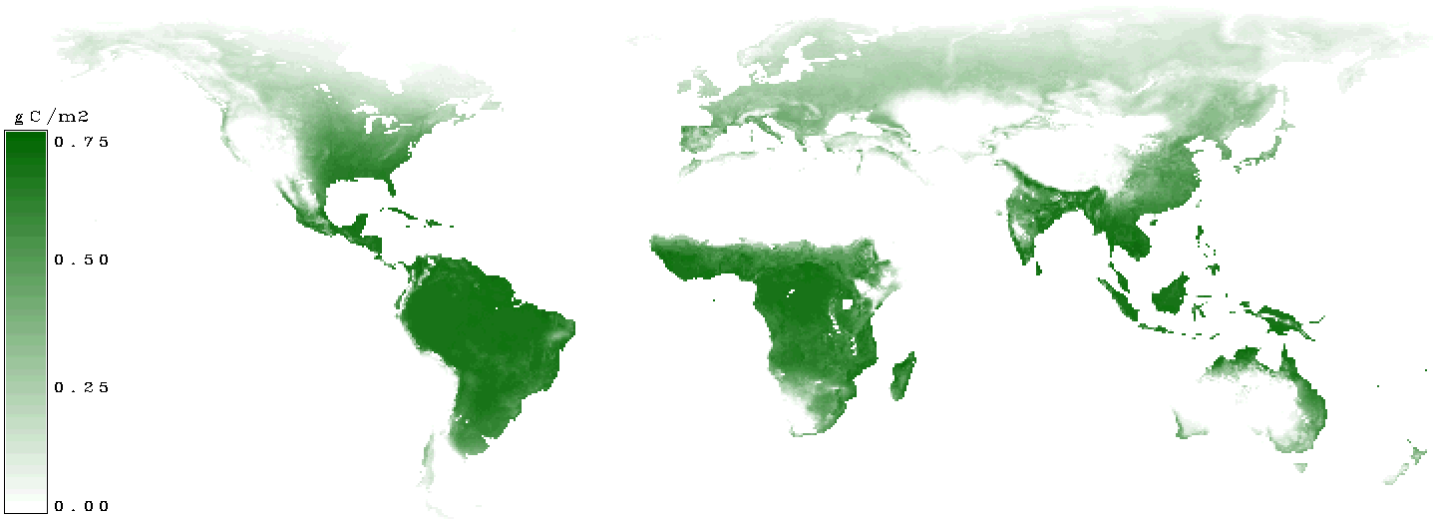
**Net Primary Production (NPP)**. Areas with a high increment have a high net primary productivity and are indicated by dark green. Sites with low productivity are indicated by light green.

In figure 11 the net primary productivity taken from [10] is shown. The values range up to 0.75 gC/m^2^/year. The highest productivity is near the equator.

**Figure 12:**
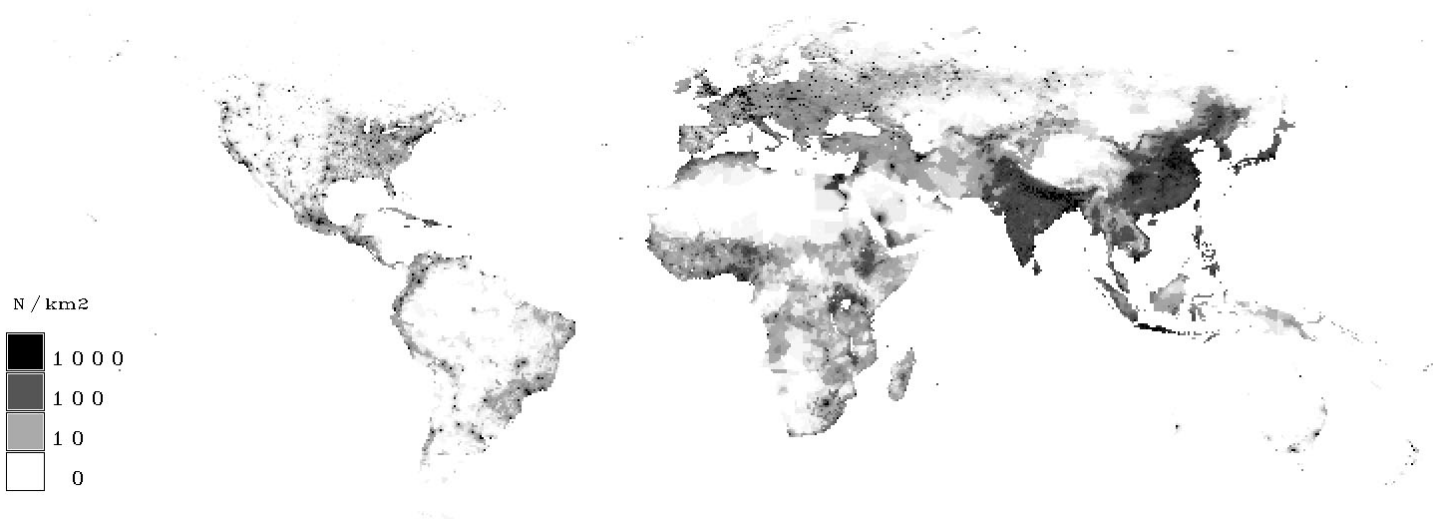
**Population density in Year 2000**. Grids with few people are given in white. A rising population density is marked by grey up to high population densities (≥1000 people/km^2^) which are indicated by black.

**Figure 13:**
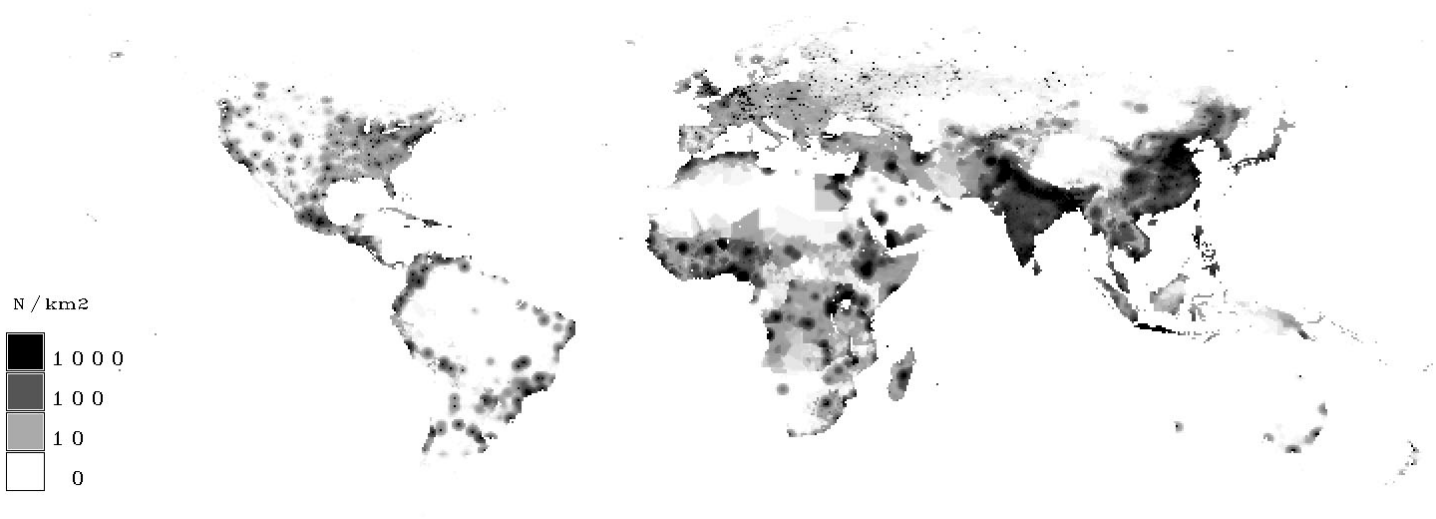
**Population density in Year 2100**. Grids with few people are given in white. A rising population density is marked by grey up to high population densities (≥1000 people/km^2^) which are indicated by black.

In figure 12 the population density in 2000 and in figure 13 in the year 2100 is shown. It can be seen, that the highest population densities are reached in India and in south-east Asia. The densities are also quite high in Europe and Little Asia, Central Africa and the coasts of America. The map of 2100 shows an increase in India and in south-east Asia.

**Figure 14:**
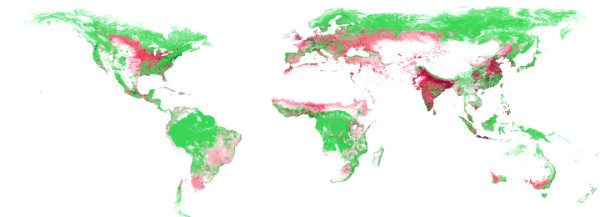
**Forest, Crop and Buildup Land cover**. Forests are shown in green, crop in red and buildup land in grey.

Figure 14 shows a map of the current forest, crop and buildup land cover. Large regions are covered by forests. Adjacent to the forests, large areas, used for crop production, can be seen.

**Figure 15:**
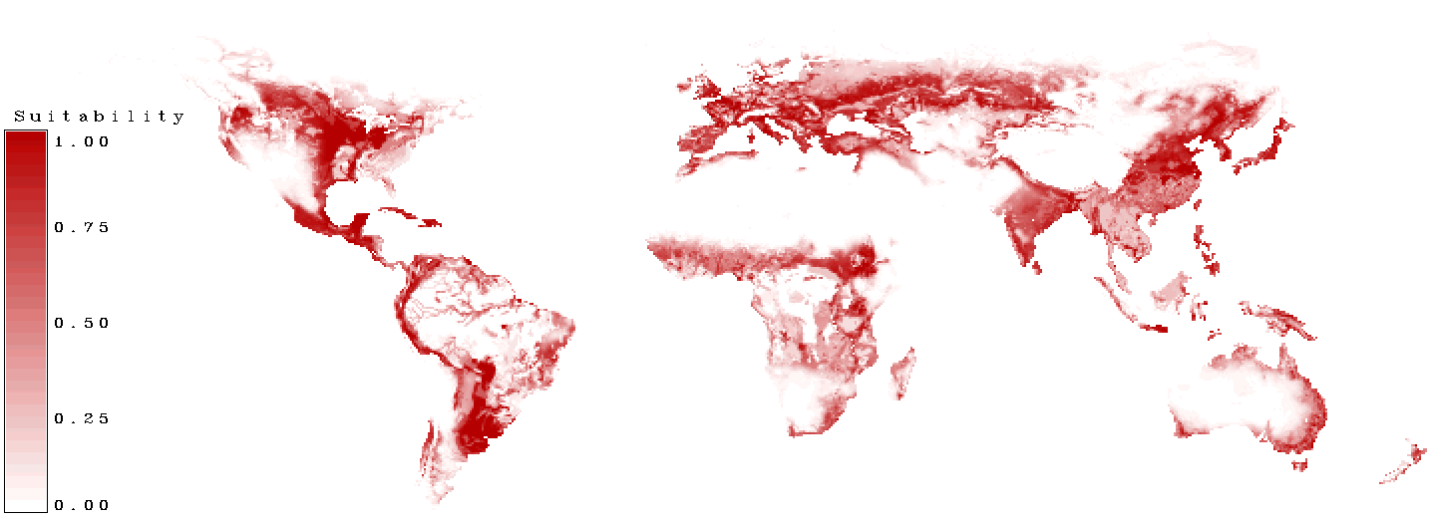
**Agriculture suitability**. High suitability for agriculture is marked in dark red. White areas are not suitable for agriculture.

In figure 15 the suitability for agriculture is shown. Most of the high suitable land is used today for crop production (see figure 14).

**Figure 16:**
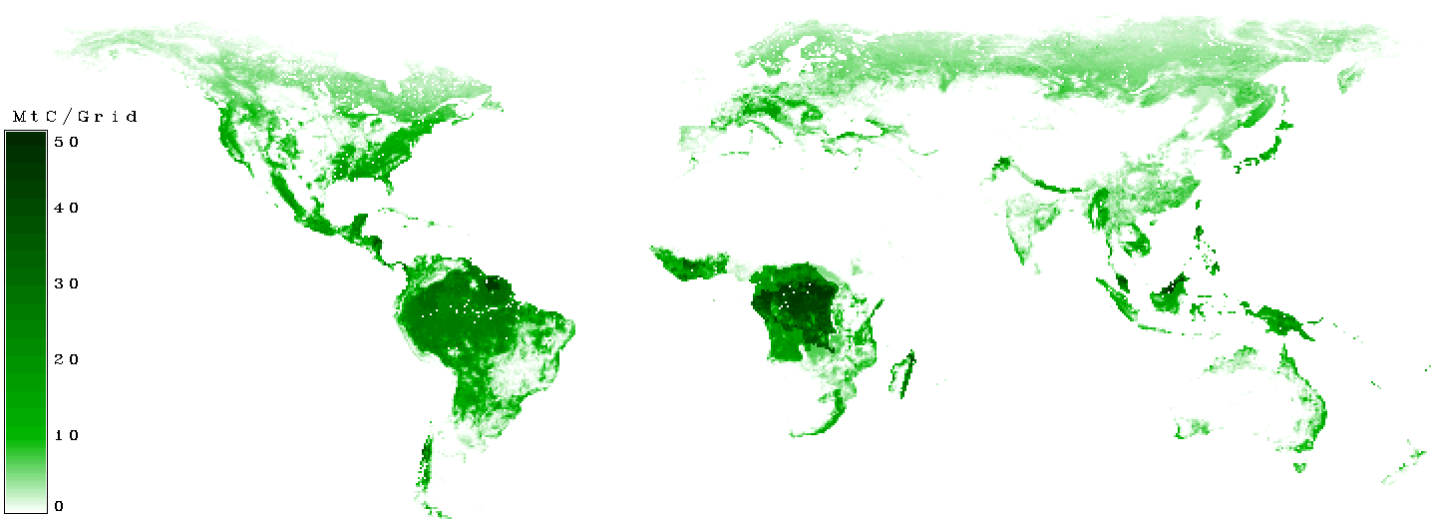
**Carbon in Forest biomass**. Regions with no carbon in forests are white. Regions with high values of carbon in forests are dark green.

Figure 16 shows the carbon in forests. It can be seen, that the highest densities are located near the tropical belt. One reason for this is, that the biomass in tropical forests is high. Note that this picture shows the tons of carbon per grid and the grid size is 0.5° × 0.5° so the grid has it's largest size near the equator.

**Figure 17:**
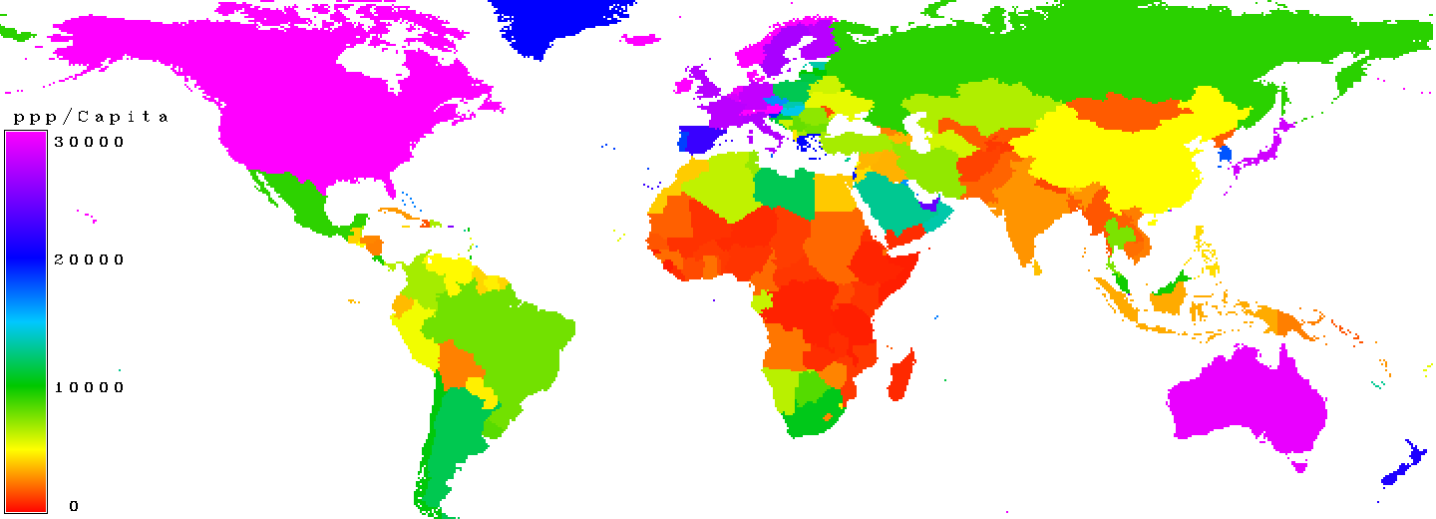
**Purchasing Power Parity (PPP)**. Countries with a low purchasing power parity are marked in red, moderate is in green, high values in blue and very high in magenta.

Figure 17 shows the purchasing power parity which was used to calculate a price-index. It can be seen that the poorest countries are in Africa and the richest in North America, Europe, Australia and Japan.

**Figure 18:**
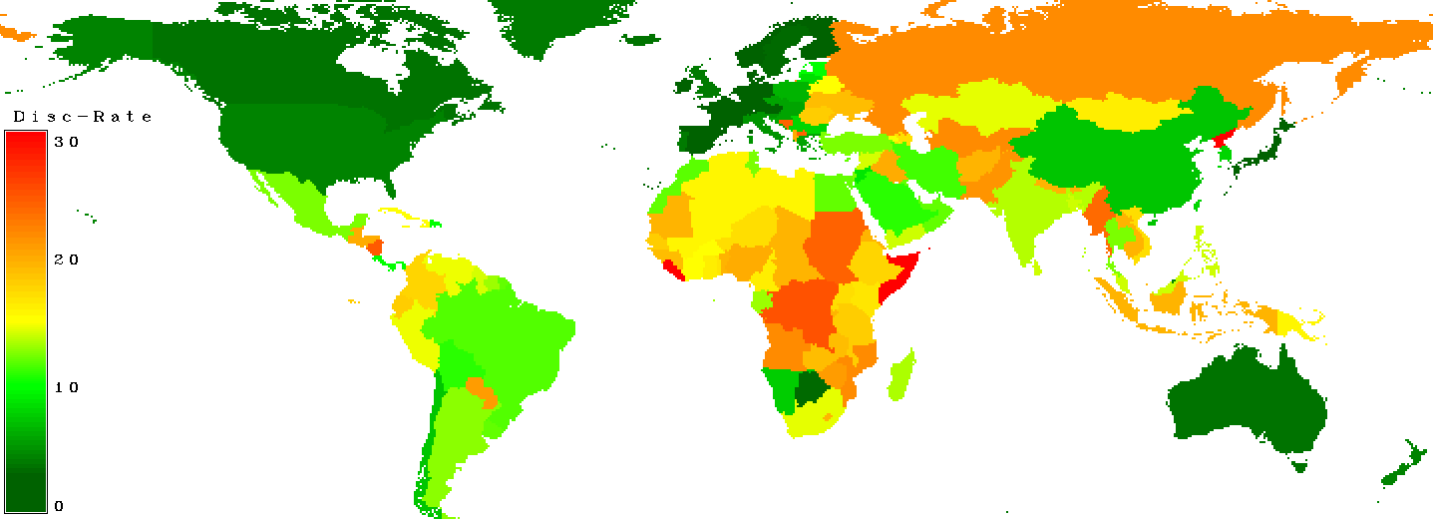
**Discount Rate**. Countries with a low discount rate are marked in dark green, moderate countries in yellow and countries with a high rate in red.

Figure 18 shows the discount-rates given in [8]. Here also the richest countries have the lowest discount rates.

**Figure 19:**
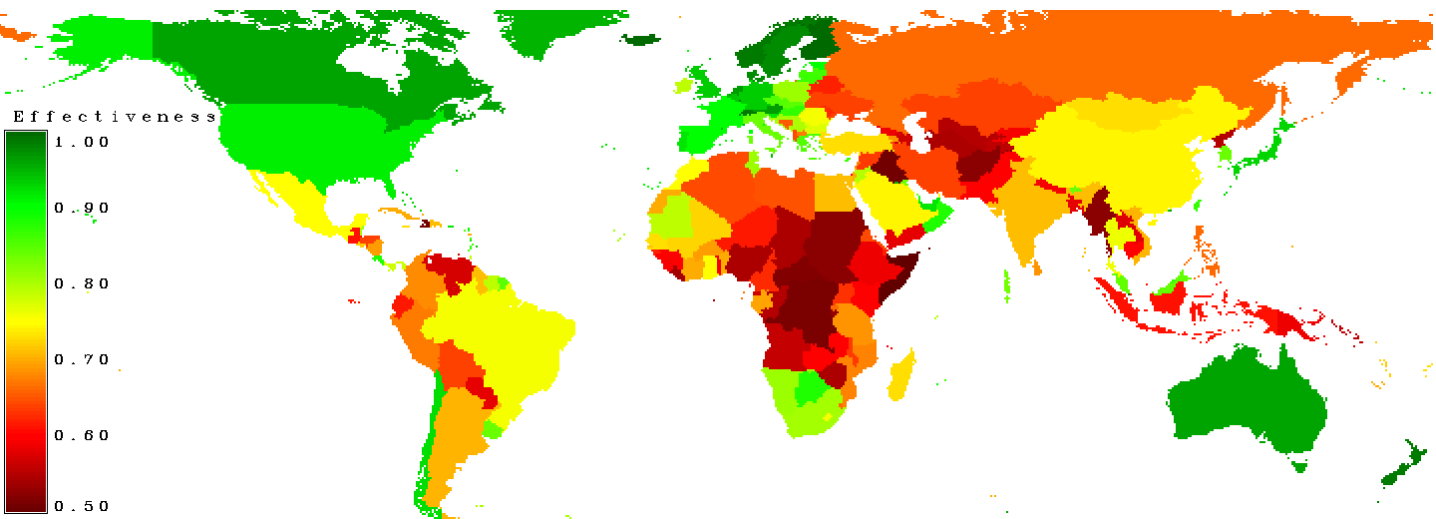
**Effectiveness (Corruption)**. Countries with high values of corruption are marked in red, moderate countries in yellow and low values in green.

Figure 19 shows the effectiveness of the carbon incentives. In low risk countries nearly all of the spent money will be used for maintaining forest sinks in risky countries not all of the money will come to the desired sink.

**Figure 20:**
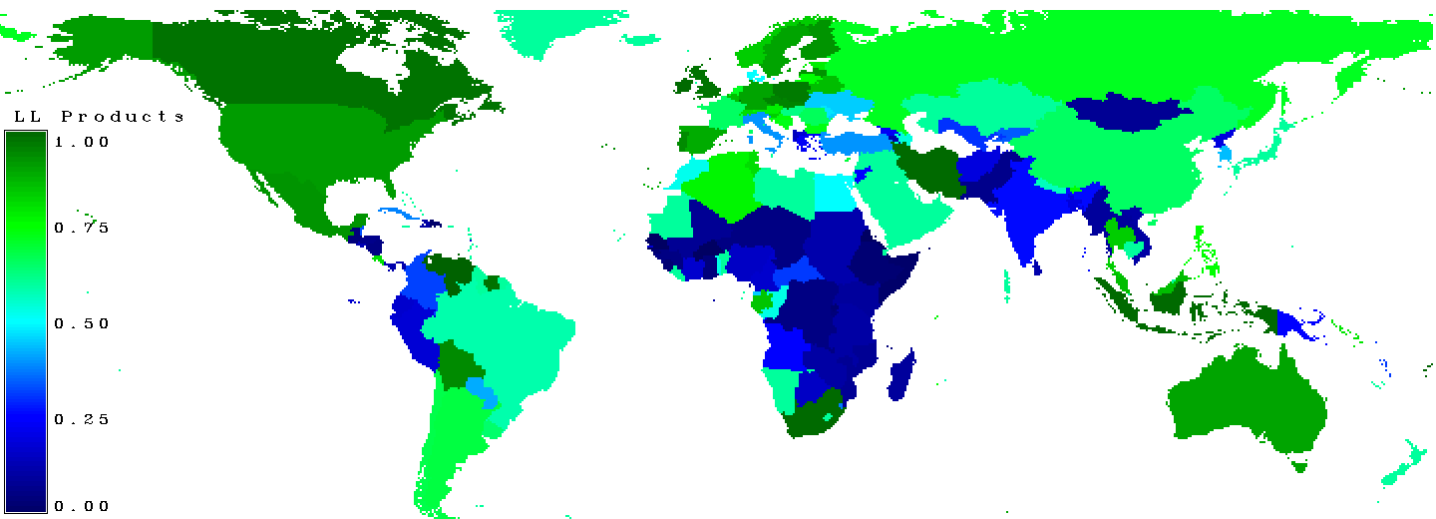
**Share of long living products**. Countries which use their wood mainly for fuel-wood are marked in blue, those who use it for sawn-wood are in green.

Figure 20 shows the proportion of harvested wood entering the long living products pool [2].

## Abbreviations

*α*_*i*_: Importance of agriculture

*γ*_*i*_: Importance of population

*ν*_*i*_: Land price level = minimum land price of reference country × price index (*px*_*i*_) [$/ha]

*ω*_*i *_Carbon uptake per year [tC/year/ha]

*θ*_*i *_: Fraction of carbon benefits in products [1]

*A*_*i*_: Net present value of agriculture [$/ha]

*AgS*_*i*_: Agricultural suitability [0–1]

*b*_*i*_: Baseline, how much carbon uptake will be if there is no forest, e.g. 0.1 [1]

*BMP*_*i*_: Biomass in Products [tC/ha]

*BM*_*i*_: Aboveground living wood biomass [tC/ha]

*B*_*i*_: Present value of carbon benefits [$/ha]

*Bul*: Share of buildup land [1]

*C*2*W*: Conversion factor form 1t Carbon to 1m^3 ^wood [m^3^/tC]

*cp*_*i*_: Planting costs [$/ha]

*cp*_*ref*_: Planting costs reference country [$/ha]

*CU*: Carbon uptake, share of NPP stored in wood [1]

*Crl*: Share of crop land [1]

*dec*_*llp*_: Decay rate of long living products e.g. 0.03 [1]

*dec*_*slp*_: Decay rate of short living products e.g. 0.5 [1]

*DV*_*i*_: Deforestation Value [$/ha]

*epc*_*i*_: Effectiv carbon price [$/tC]

*f*_*i*_: Net present value of forestry for one rotation period [$/ha]

*F*_*i*_: Net present value of forestry [$/ha]

*F*_*s*_: Actual share of forest [0–1]

*F*_*dec*_: Decrease of the forest share

*fr*_*i*_: Frequency of incentives money payment [Years]

*frac*_*llp*_: Fraction of long living products e.g. 0.5 [0–1]

*frac*_*sb*_: Fraction of slash burned area e.g. 0.9 [0–1]

*frac*_*slp*_: Fraction of short living products e.g. 0.5 [0–1]

*Fs*: Forest area share [0–1]

*Fs*_*year*_: Forest share of a certain year [1]

*fsx*_*year*_: Theoretical forest share of a certain year [1]

*Ft*_*dec *_: Theoretical decrease of the forest share

*GDP*: Gross domestic product [$_1995_/Person]

*H*_*i*_: Hurdle e.g. 1.5 [1]

*HL*_*i*_: Harvesting losses e.g. 0.2 [1]

*i*: Grid number

*leak*_*i*_: Factor of money which will in real reach the forest [1]

*IP*_*i*_: Incentive payment [$/ha]

*MAI*_*i*_: Mean annual wood volume increment [m^3^/ha]

*NPP*_*i*_: Net primary production [tC/ha/year]

*pc*_*i*_: Carbon price [$/tC]

*pca*_*i*_: Incentives carbon price [$/tC/*fr*_*i*_]

*Pd*_*i*_: Population density [People/km^2^]

*PL*_*max*_: Maximal land price of reference country × price index (*px*_*i*_) [$/ha]

*PL*_*min*_: Minimal land price of reference country × price index (*px*_*i*_) [$/ha]

*PPP*_*i*_: Purchasing power parity [$]

*PPP*_*ref*_: Purchasing power parity of reference country [$]

*pr*_*i*_: Ratio of area planted [0–1]

*pw*_*i*_: Stumpage wood price [$/m^3^]

*pw*_*max*_: Maximum revenue of wood, e.g. 35$/fm [$/fm]

*Pw*_*min*_: Minimum revenue of wood, e.g. 5$/fm [$/fm]

*px*_*i*_: Price index [1]

*r*: Discount rate [e.g. 0.05]

*R*_*i*_: Rotation interval length [years]

*SAgS*_*i*_: Standardized agricultural suitability [1-10]

*SFs*: Standardized not forest area share [1-10]

*SPd*: Standardized population density [1-10]

*V*_*i*_: Harvest wood volume [m^3^]

*x*_*i*_: Theoretical decrease of the forest share if *Fs*_*i *_> 0 ∧ *AgS*_*i *_> 0

## Competing interests

The author(s) declare that they have no competing interests.

## Authors' contributions

Georg Kindermann has developed the deforestation rate model, implemented the whole model, collected some data sources and organized them to be used for the implementation, runs the simulations, created figures and tables and wrote a first draft of the paper.

Michael Obersteiner developed the core model describing the forest value, agricultural value and decision of deforestation, worked on the paper, introduced the maximum tax income and contributed to the payment possibilities.

Ewald Rametsteiner contributed to the carbon price and incentives model and their practical implementation, worked on the paper and brought in many background informations.

Ian McCallum collected and organized the data source and produced some figures of the paper.
